# A review of epilepsy detection and prediction methods based on EEG signal processing and deep learning

**DOI:** 10.3389/fnins.2024.1468967

**Published:** 2024-11-15

**Authors:** Xizhen Zhang, Xiaoli Zhang, Qiong Huang, Fuming Chen

**Affiliations:** ^1^Medical Support Center, No. 940 Hospital of the Joint Logistics Support Force of the Chinese People’s Liberation Army, Lanzhou, China; ^2^Gansu University of Traditional Chinese Medicine, Lanzhou, China

**Keywords:** preprocessing, feature extraction, epilepsy detection, epilepsy prediction, deep learning

## Abstract

Epilepsy is a chronic neurological disorder that poses significant challenges to patients and their families. Effective detection and prediction of epilepsy can facilitate patient recovery, reduce family burden, and streamline healthcare processes. Therefore, it is essential to propose a deep learning method for efficient detection and prediction of epileptic electroencephalography (EEG) signals. This paper reviews several key aspects of epileptic EEG signal processing, focusing on epilepsy detection and prediction. It covers publicly available epileptic EEG datasets, preprocessing techniques, feature extraction methods, and deep learning-based networks used in these tasks. The literature is categorized based on patient independence, distinguishing between patient-independent and non-patient-independent studies. Additionally, the evaluation methods are classified into general classification indicators and specific epilepsy prediction criteria, with findings organized according to the prediction cycles reported in various studies. The review reveals several important insights. Despite the availability of public datasets, they often lack diversity in epilepsy types and are collected under controlled conditions that may not reflect real-world scenarios. As a result, signal preprocessing methods tend to be limited and may not fully represent practical conditions. Feature extraction and network designs frequently emphasize fusion mechanisms, with recent advances in Convolutional Neural Networks (CNNs) and Recurrent Neural Networks (RNNs) showing promising results, suggesting that new network models warrant further exploration. Studies using patient-independent data generally produce better results than those relying on non-patient-independent data. Metrics based on general classification methods typically perform better than those using specific epilepsy prediction criteria, though future research should focus on the latter for more accurate evaluation. Epilepsy prediction cycles are typically kept under 1 h, with most studies concentrating on intervals of 30 min or less.

## 1 Introduction

Epilepsy is a chronic neurological disorder affecting over 50 million people worldwide, according to the World Health Organization. It can impact individuals of all ages and is characterized by recurrent seizures, which result from sudden, abnormal discharges of neurons in the brain (Pelkonen et al., 2020). These seizures can vary widely in severity and presentation, ranging from brief lapses in awareness to full-body convulsions. The underlying causes of epilepsy are diverse, including genetic, infectious, structural, immune, metabolic, and sometimes unknown factors (Scheffer et al., 2017). The primary treatments for epilepsy include medication and surgery, but these interventions are not always effective (Perucca, 2021). Approximately 30% of patients continue to experience recurrent seizures despite treatment (Liu et al., 2021). These seizures can lead to a range of symptoms, including loss of consciousness, muscle twitching, and difficulty breathing (World Health Organization, 2024). In severe cases, seizures can result in falls, injury, and even drowning, posing significant risks to life. Additionally, the social implications of epilepsy, such as the potential for discrimination and the stigma associated with the condition, can exacerbate psychological distress. Studies have shown that individuals with epilepsy, especially children and adolescents, are more likely to suffer from anxiety and depression due to the chronic and unpredictable nature of their condition (Puka et al., 2017). EEG, which records the brain’s electrical activity, is a crucial tool in diagnosing and managing epilepsy. However, analyzing EEG data is time-consuming and requires the expert of trained neurologists. The accuracy of diagnosis heavily depends on the neurologist’s experience and skill. This traditional approach has its limitations: particularly given the complexity and volume of EEG data that must be reviewed (Chandani and Kumar, 2018). In recent years, the development of deep learning techniques has offered new possibilities for improving epilepsy detection and prediction. These advanced algorithms can process and analyze large volumes of EEG data more efficiently than traditional methods, assisting neurologists in diagnosing epilepsy and predicting seizures more accurately. By providing timely warnings, these technologies can help patients take preventive measures, reducing the physical and psychological impact of seizures. This paper provides a comprehensive review of the current research on epileptic EEG signal detection and prediction using deep learning methods. EEG has the advantages of simplicity, safety, high temporal resolution, and high utilization, making it a key tool in diagnosing epilepsy (Wei et al., 2021; Ein Shoka et al., 2023). Given this prevalence, this paper concentrates on reviewing literature specifically related to EEG signals in the context of epilepsy. We will explore four key areas: the nature of epileptic EEG data, preprocessing techniques, feature extraction methods, and deep learning-based detection and prediction algorithms. This review also addresses gaps in existing literature, including issues related to data partitioning, model evaluation methods, and prediction timeframes, offering a more detailed perspective on the state of the field.

## 2 Epileptic EEG signals

### 2.1 Partitioning of epileptic EEG signal states

**Figure 1:**
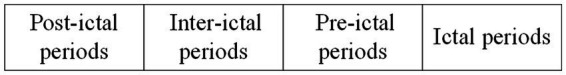
Four states of epileptic EEG signals.

EEG signals of an epileptic patient can be categorized into four states: Post-ictal state, Inter-ictal state: Pre-ictal state and Ictal state. The pre-ictal state is the state minutes before the actual occurrence of the seizure. The ictal state is the state actual occurrence of the seizure. The post-ictal state is the state after the seizure has passed. The inter-ictal state is the state between post-ictal state and preictal state. Signals during ictal and inter-ictal periods are often used as data for detecting the occurrence of epilepsy. Signals during pre-ictal and inter-ictal periods are often used as data to predict whether epilepsy will occur or not (Aslam et al., 2022). Figure 1 illustrates the four states of epileptic EEG signals.

### 2.2 Data presentation

The 10–20 International Standard Electrode Placement System, established by the International Federation of Clinical Neurophysiology, is widely recognized as the standard method for electrode placement in EEG data acquisition (Maillard and Ramantani, 2017). This system is employed by most publicly available epileptic datasets, ensuring consistency and reliability in data collection. In this section, we will discuss commonly used datasets in epilepsy research. Most of these datasets are freely accessible, with the exception of the Bonn dataset, which requires a purchase. These datasets typically organize epileptic EEG data on a patient-by-patient basis, with each patient’s data stored in separate folders. This organization leads to two primary methods of data segmentation for epilepsy detection and prediction: patient-independent and non-patient-independent methods.

**Figure 2:**
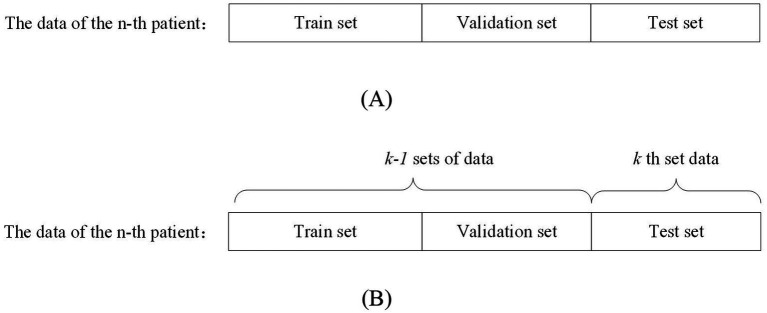
Schematic diagram of patient-independent data segmentation.

Patient-independent methods refer to data from a particular patient as training, validation, and testing data for the model. This data partitioning approach can be further divided into two types: segment-based approach and event-based approach. Segment-based approach means that the data of a particular patient is divided into training, validation, and testing sets. The event-based approach means: dividing the data of a patient into k groups according to the number of seizures, using the k-1 group of data as the training and validation set, and the kth group of data as the test set. This approach generally yields more accurate evaluation results compared to non-patient-independent methods. However, it requires a substantial amount of patient-specific data, which must be recorded over different seizure periods to effectively train the model for detecting or predicting seizures in that particular patient. Figure 2A shows the segment-based data partitioning method and Figure 2B shows the event-based data partitioning method.

**Figure 3:**
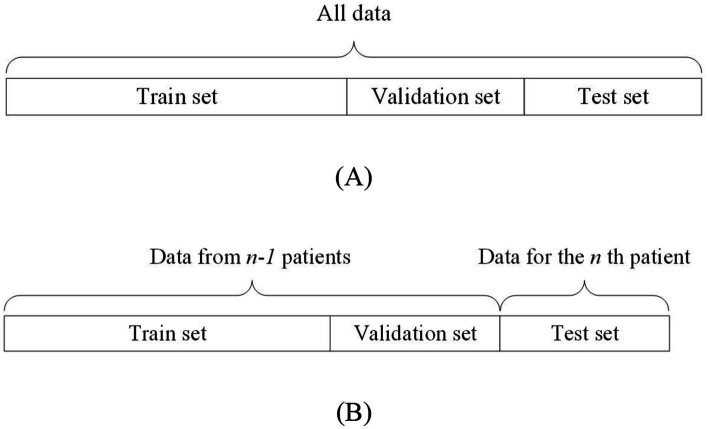
Schematic diagram of non-patient independent data segmentation.

On the other hand, non-patient-independent methods refer to the use of all patients’ data as training, validation, and testing data for the model. This data partitioning can be further divided into two types: the all-patient approach and the cross-patient approach. The difference is as follows: the all-patient approach divides data from all patients into training, validation, and testing sets. The cross-patient approach refers to dividing the data of n-1 patients as the training set and validation set, and the remaining patient data as the test set. Figure 3A shows the all-patient data division method and Figure 3B shows the cross-patient data division method.

**Table 1:** Information on publicly available epileptic EEG datasets.

Data sets	Subjects	Electrode placement system	sampling frequency (Hz)	Detection	Prediction	Patient independence	Non-patient independence	Signal acquisition location
CHB-MIT (Shoeb, 2009)	23	10–20	256	Yes	Yes	Yes	Yes	Scalp
Siena (Detti, 2020; Goldberger et al., 2000)	14	10–20	512	Yes	Yes	Yes	Yes	Scalp
Freiburg (Zhang and Parhi, 2016)	21	–	256	Yes	Yes	Yes	Yes	iEEG
SWEC-ETHZ iEEG (The SWEC-ETHZ iEEG Database and Algorithms Overview, 2024; Burrello et al., 2018; Burrello et al., 2019)	18	-	512/1024	Yes	Yes	Yes	Yes	iEEG
	16		512	Yes	No	Yes	Yes	
New Delhi (Swami et al., 2016)	10	10–20	200	Yes	Yes	No	Yes	Scalp
Helsinki (Stevenson, 2019)	79	10–20	256	Yes	Yes	Yes	Yes	Scalp
Bonn (Andrzejak et al., 2001)	10	10–20	173.61	Yes	No	No	Yes	Scalp, iEEG
TUH (Harati et al., 2014)	115	–	250/256/400	Yes	No	No	Yes	–
Kaggle (Brinkmann et al., 2016; Zhao et al., 2022; Bbrinkm Sbaldassano Will Cukierski, 2014)	7	–	400/5000	No	Yes	Yes	Yes	iEEG
	12	–	400/500 ~ 5,000	Yes	No	Yes	Yes	
BERN-BARCELONA (Xin et al., 2022)	5	10–20	512/1024	Yes	No	No	Yes	iEEG
Epileptic EEG Dataset (Nasreddine, 2021)	6	10–20	500	Yes	Yes	Yes	Yes	Scalp
EPILEPSIAE (Ihle et al., 2012; Klatt et al., 2012)	275	–	–	Yes	Yes	Yes	–	Scalp, iEEG

## 3 Techniques for pre-processing EEG signals in epilepsy

During EEG acquisition, artifacts such as electrocardiogram (ECG) signals, electromyogram (EMG) signals, and thermal noise can contaminate the data. Effective preprocessing of these artifacts is essential for improving the accuracy of epilepsy detection and prediction models. Several techniques are widely employed in the preprocessing of epileptic EEG signals, including thermal noise reduction, artifact removal, and data enhancement methods.

### 3.1 Thermal noise processing of EEG signals

Electromagnetic interference in the ambient environment and thermal noise inherent in the device can severely damage the low-amplitude EEG signal. The raw EEG signal is highly non-stationary and dynamic, and the scalp EEG itself is small in amplitude, so it is easily affected by high-frequency interferences as well as 50 Hz or 60 Hz signals (Lakehal and Ferdi, 2024; Wang et al., 2024). A 50 Hz or 60 Hz notch filter is commonly used to remove the industrial frequency noise (Raghu et al., 2020). High-frequency noise is filtered out using a high pass filter, low pass filter, band pass filter, etc (Liu et al., 2022).

### 3.2 Removal of artifacts in EEG signals

To address thermal noise and artifacts in EEG data, various signal processing methods have been developed. Techniques such as Wavelet Transform (WT), Empirical Mode Decomposition (EMD), and Blind Source Separation (BSS) are commonly employed to identify and remove these artifacts.

WT denoising works by using a basis function to compute the wavelet coefficient that represent the degree of similarity between the basis function and the original signal. Noise is removed by setting a threshold for these coefficients; those below the threshold are discarded, and the remaining coefficients are used to reconstruct the denoised signal. For example, Zhou et al. (2021) proposed a dynamic thresholding method based on Discrete Wavelet Transform (DWT) for artifact removal, which has proven effective in enhancing EEG signal quality. Similarly, Yedurkar and Metkar (2020) applied DWT with adaptive filtering to remove low-frequency physiological artifacts while retaining more useful signal components.

However, the basis function in wavelet denoising is set manually and lacks adaptability. This limitation can be addressed by EMD, which decomposes any complex signal into multiple Intrinsic Mode Functions (IMFs) at different frequencies. A denoised signal can be obtained by setting a threshold to discard inappropriate IMFs and reconstructing the remaining ones. Parija et al. (2020) processed EEG signals by selecting the first four high-frequency IMFs from EMD, although the selection criteria lack a strong theoretical basis. Moctezuma and Molinas (2020) further refined EMD by filtering out noisy IMFs using Minkowski distance, while Karabiber Cura et al. (2020) applied energy, correlation, and other statistical measures to screen suitable IMFs for both EMD and Ensemble Empirical Mode Decomposition (EEMD). Hassan et al. (2020) obtained clean EEG signals using Complete Ensemble Empirical Mode Decomposition with Adaptive Noise (CEEMDAN) and NIG parameters. Bari and Fattah (2020) concluded that extracting the first IMF components is the most desirable by comparing the amplitudes and frequencies of IMFs obtained after CEEMDAN decomposition. Variational Modal Decomposition (VMD) offers an alternative approach, solving the problem of mode mixing in EMD and reducing computational complexity, as demonstrated by Liu et al. (2022), who removed noise by correlating VMD functions with the original signal. Peng et al. (2021) proposed the Elastic Variational Mode Decomposition (EVMD) algorithm, which is able to capture the center frequency variations of EEG signals in various frequency band segments compared to VMD, thus improving the experimental performance.

However, EMD has limitations, including mode mixing, noise introduction, and high computational complexity. These drawbacks can be addressed by BSS. BSS operates on the principle that a signal can be represented as a linear combination of different source signals. By decomposing the signal into multiple source components, discarding the noisy ones, and reconstructing the remaining components, a denoised signal can be obtained. Classical BSS techniques include Canonical Correlation Analysis (CCA), Principal Component Analysis (PCA), and Independent Component Analysis (ICA). While CCA and PCA are effective for analyzing linear signals, ICA is better suited for nonlinear signals, making it a more commonly used method for artifact removal in EEG data. Islam et al. (2020) introduced an advanced ICA variant, Infomax ICA, which showed improved reliability in separating components based on mutual information. Becker et al. (2015) enhanced ICA with a penalized semialgebraic deflation algorithm, reducing computational complexity while maintaining performance. Beyond conventional approaches, several innovative methods have been developed for denoising epileptic EEG signals. Sardouie et al. (2014) introduced two semi-blind source separation techniques based on time-frequency analysis: Time-Frequency Generalized Eigenvalue Decomposition (TF-GEVD) and Time-Frequency Denoised Source Separation (TF-DSS). These methods were shown to outperform traditional techniques. Qiu et al. (2018) proposed the Denoising Sparse Autoencoder (DSAE), an advanced deep neural network that combines the strengths of sparse and denoising autoencoders. By enforcing sparsity in the hidden layers, this method efficiently represents EEG signals, particularly when dealing with non-smooth, noisy data. The DSAE’s ability to learn robust representations of the underlying EEG signals makes it a valuable tool for improving the accuracy of seizure detection. Lopes et al. (2021) explored a deep learning approach by developing a Deep Convolutional Neural Network (DCNN) model for artifact removal in EEG data. This model is particularly effective in handling nonlinear signal sources and removing various artifacts, including those caused by eye blinks, muscle activity, and channel motion. Despite its complexity, the DCNN model offers significant improvements in denoising performance.

However, BSS requires a reference waveform to effectively remove noise by linking it with multiple source signal waveforms. Recognizing that each denoising method has its strengths and limitations, researchers have also explored hybrid approaches to improve the purity of EEG signals. Du et al. (2024) employed a combination of CEEMDAN and Continuous Wavelet Transform (CWT) for joint denoising of epileptic EEG signals. This approach further improves signal quality by integrating the benefits of both CEEMDAN and CWT, offering a more comprehensive noise reduction strategy. Please refer to Table 2 for details.

**Table 2:** Denoising methods for epileptic EEG signals.

Author	Dataset	Denoise methods	Type	Result	Advantages	Disadvantages
Zhou et al. (2021)	Non-public	DWT, dynamic thresholding	DWT	precision:86.8% sensitivity: 82.7%	Window is Adjustable (Chen et al., 2024)	Basis functions are not adaptive (Sharma, 2017; Huang et al., 1998)
Yedurkar and Metkar (2020)	Non-public CHB-MIT	DWT, adaptive filtering		accuracy: 86.66% Precision:88.88%		
Parija et al. (2020)	Bonn	EMD	EMD	Accuracy:100%	Basis functions are adaptive (Das et al., 2024)	modal aliasing (Song et al., 2023) High time complexity
Moctezuma and Molinas (2020)	CHB-MIT	Minkowski Distance, EMD		accuracy: 93%		
Karabiber Cura et al. (2020)	Non-public	EEMD		1.5% improvement in classification accuracy	Reducing modal aliasing	Noise residue (Lan et al., 2024) High time complexity
Hassan et al. (2020)	Bonn	CEEMDAN, NIG parameters		Over 97% accuracy, sensitivity, specificity	Reducing noise residue	High time complexity
Bari and Fattah (2020)	Bonn	CEEMDAN		Accuracy reduced by 1%, computational complexity and time complexity reduced		
Liu et al. (2022)	Bonn Freiburg	Correlation,VMD		Sensitivity and specificity of more than 95%	Reduced modal aliasing and reduced computational complexity	Slow parameter selection and poor generalization
Peng et al. (2021)	BERN-BARCELONA	EVMD		Accuracy, sensitivity and specificity increased by more than 3%.		
De Vos et al. (2011)	Non-public	ICA	BSS	The number of false alarms has been reduced by almost four times	No need to know the signal artifact type (Uddin et al., 2023), Lower time complexity than EMD	Reference signals required (Xu et al., 2024)
Islam et al. (2020)	Non-public	Infomax ICA		Accuracy can be improved by 24%		
Becker et al. (2015)	Non-public	deflation ICA		Reduce computational complexity by a factor of 10		
Sardouie et al. (2014)	Non-public	TF-GEVD, TF-DSS		Better results than CCA and ICA		
Qiu et al. (2018)	Bonn	DSAE	Deep learning	Performance improved by 8.19%	Less residual noise	High computational complexity, High time complexity
Lopes et al. (2021)	EPILEPSIAE	DCNN		Evaluation indicators are better than Infomax ICA-MARA		
Jana et al. (2022)	CHB-MIT Bonn	DWT-EMD	Fusion methods	Accuracy can be improved by 19.58%	Less residual noise	High time complexity
Du et al. (2024)	New Delhi	CEEMDAN-CWT		The signal-to-noise ratio (SNR) can be increased by 1.0567 dB and the root mean square error (RMSE) can be reduced by 0.1045		

### 3.3 Data enhancement

In addition to artifact removal, data enhancement techniques are crucial for improving the quality of EEG signals, especially when dealing with small or imbalanced datasets. Data augmentation methods, such as flipping, windowing, and adding noise, are commonly used to artificially expand datasets (Wan et al., 2023). Palanisamy and Rengaraj (2024) employed these techniques by increasing the amplitude and applying random transformations to EEG signals, thereby enhancing the robustness of deep learning models.

Generative Adversarial Networks (GANs) represent a more sophisticated approach to data augmentation. Initially proposed by Goodfellow et al. (2014), GANs generate synthetic data that can balance training datasets, as demonstrated by Gao et al. (2022) with their GAN-based model for seizure detection. Temporal Generative Adversarial Networks (TGAN) and Wasserstein Generative Adversarial Networks (WGANs) have also been employed to address temporal dependencies and gradient vanishing issues in EEG data, respectively. Nasiri and Clifford (2021) propose a Generative Transferable Adversarial Network (GTAN) that generates transferable adversarial features to address the performance degradation of traditional algorithms due to inter-individual variation in EEG data. A comparison of each method is shown in Table 3.

**Table 3:** A comparison of each method.

Author	Net	Result
Gao et al. (2022)	GAN	More than 20% higher sensitivity
Ganti et al. (2022)	TGAN	18.5% improvement in accuracy
Wei et al. (2019)	WGAN	Specificity increased by more than 3%
Nasiri and Clifford (2021)	GTAN	More than 5% improvement in accuracy and more than 7% improvement in specificity

## 4 Feature extraction techniques for epileptic EEG signals

Feature extraction can streamline and organize underlying patterns, making them more manageable. Although neural networks are capable of automatically identifying useful features from raw data, well-engineered features can simplify problem-solving. This approach often requires less data and fewer resources to achieve effective results (Francois, 2022). Feature extraction in EEG signal analysis involves time-domain features, frequency-domain features, time-frequency domain features, and nonlinear dynamics features. Time-domain features provide a general overview of the signal’s distribution but do not capture frequency-related information. Conversely, frequency-domain features convert signals from the time domain to the frequency domain, enabling the extraction of frequency-specific information. Time-frequency domain methods offer a more nuanced approach by representing spectral signals over time, combining both temporal and spectral analyses (Pham, 2021; Qin et al., 2024). Nonlinear dynamics, on the other hand, are used to quantify the chaotic nature of EEG signals, offering insights into their complexity (Yan et al., 2022).

Sun and Chen (2022) utilized entropy-based features, such as Sample Entropy (SampEn), Permutation Entropy (PermEn), and Fuzzy Entropy (FuzzyEn), both individually and in combination, to form three-dimensional feature vectors. Their findings indicated that combining SampEn, PermEn, and FuzzyEn produced the highest accuracy and recall rates. However, this approach did not incorporate time or frequency domain information. Fei et al. (2017) addressed this limitation by employing the Fractional Fourier Transform (FrFT), the adaptive maximal Lyapunov exponent, and its energy, which captured both the chaotic and frequency domain characteristics of epileptic EEG signals. Wang et al. (2019) Extracting features of the signal using Teager energy entropy, curve length and Teager-Huang transform results in better results than ordinary energy entropy. Zhang et al. (2024) took a more comprehensive approach by extracting features using DWT, Power Spectral Density (PSD), standard deviation, band energy, and FuzzyEn. While this method effectively gathered diverse feature information, it also introduced redundancy and increased computational complexity. To mitigate these issues, Zhang et al. (2018) generated 2,794 features for each sample using multiple feature extraction methods. These features were evaluated by a series of three consecutive selection algorithms, i.e., VarA, iRFE, and BackFS, to screen important features. Table 4 presents a summary of various feature extraction methods employed in the analysis of epileptic EEG signals.

**Table 4:** Feature extraction methods for epileptic EEG signals.

Author	Feature extraction methods	Feature extraction type	Result
Sun and Chen (2022)	SampEn, PermEn, FuzzyEn	Nonlinear dynamics	Accuracy: 6.9% improvement, recall: 4.69% improvement
Fei et al. (2017)	FrFT, energy	Time-frequency domain	Accuracy, sensitivity, and specificity: 2.5% improvement
	Adaptive Maximum Lyapunov	Nonlinear dynamics	
Zhang et al. (2024)	DWT	Time-frequency domain	Accuracy, sensitivity, and specificity of 99% or higher
	FuzzyEn	Nonlinear dynamics	
	Standard deviation	Time domain	
	band energy, PSD	Frequency domain	
Wang et al. (2019)	Teager energy entropy, curve length	Time domain	Results are better than normal energy entropy
	Teager-huang transform	Time-frequency domain	
Zhang et al. (2018)	Statistical Moments, Root Mean Square: eak, Minimum, Maximum, peak to Average Power Ratio, Form Factor, Hurst, Fischer Information	Time domain	Accuracy: 25.65% improvement
	Higuchi fractal dimension, Petrosian fractal dimension, Mandelbrot fractal dimension, Hjorth parameter, SampEn, PermEn, Spectral entropy, Singular Value Decomposition (SVD) entropy	Nonlinear dynamics	

## 5 Algorithms for detection and prediction of epileptic EEG signals

After preprocessing and feature extraction on the data, the processed signal is then fed into the network model for deep feature extraction and classification.

### 5.1 Assessment of indicators

In evaluating deep learning models for epileptic EEG signal detection and prediction, specific assessment metrics are employed to measure performance effectively.

For seizure detection, the evaluation is typically straightforward, relying on standard classification metrics such as Accuracy, Sensitivity, Specificity, Precision, F1 Score, Receiver Operating Characteristic (ROC) curve, Area Under the ROC Curve (AUC), and False Positive Rate (FPR). These metrics provide a clear measure of how well the model distinguishes between seizure and non-seizure events.

**Figure 4:**
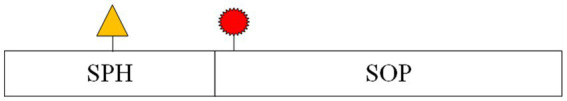
Schematic diagram of SPH and SOP concepts.

In contrast, seizure prediction models are designed to differentiate between inter-ictal (periods between seizures) and pre-ictal (periods just before a seizure) states, which requires a more nuanced approach. While prediction models often use the same metrics as detection models, a specialized set of criteria—known as the Epilepsy Prediction Evaluation Criteria—was introduced by Maiwald et al. (2004). This method includes several key metrics: Maximum False Positive Rate (FPRmax): The highest allowable prediction error rate within a given time interval. Seizure Onset Period (SOP): The timeframe during which a seizure is expected to occur following a prediction. Seizure Prediction Horizon (SPH): The time interval between the prediction and the SOP. Figure 4 provides a schematic representation of SPH and SOP.

**Figure 5:**
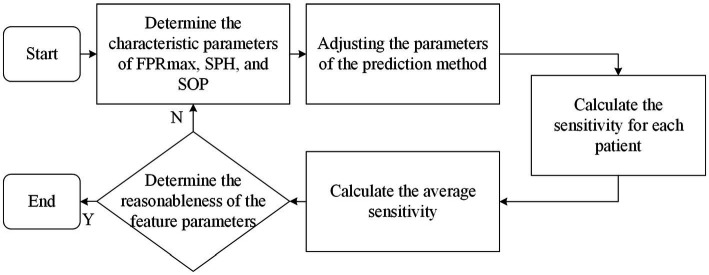
Flowchart for determining FPRmax, SPH and SOP.

The process for applying these criteria begins with setting initial values for FPRmax, SPH, and SOP. The model’s parameters are then adjusted to ensure that the false prediction rate for each patient remains within the set FPRmax. The sensitivity for each patient is calculated, and the average sensitivity across all patients is determined. This process is repeated iteratively to establish reasonable ranges for FPRmax, SPH, and SOP, as shown in Figure 5. This methodology builds on earlier work by Osorio et al. (1998), who emphasized the importance of considering both FPR and sensitivity in prediction models. Notably, there is a trade-off between the FPR and sensitivity.

### 5.2 Convolutional neural networks

**Figure 6:**
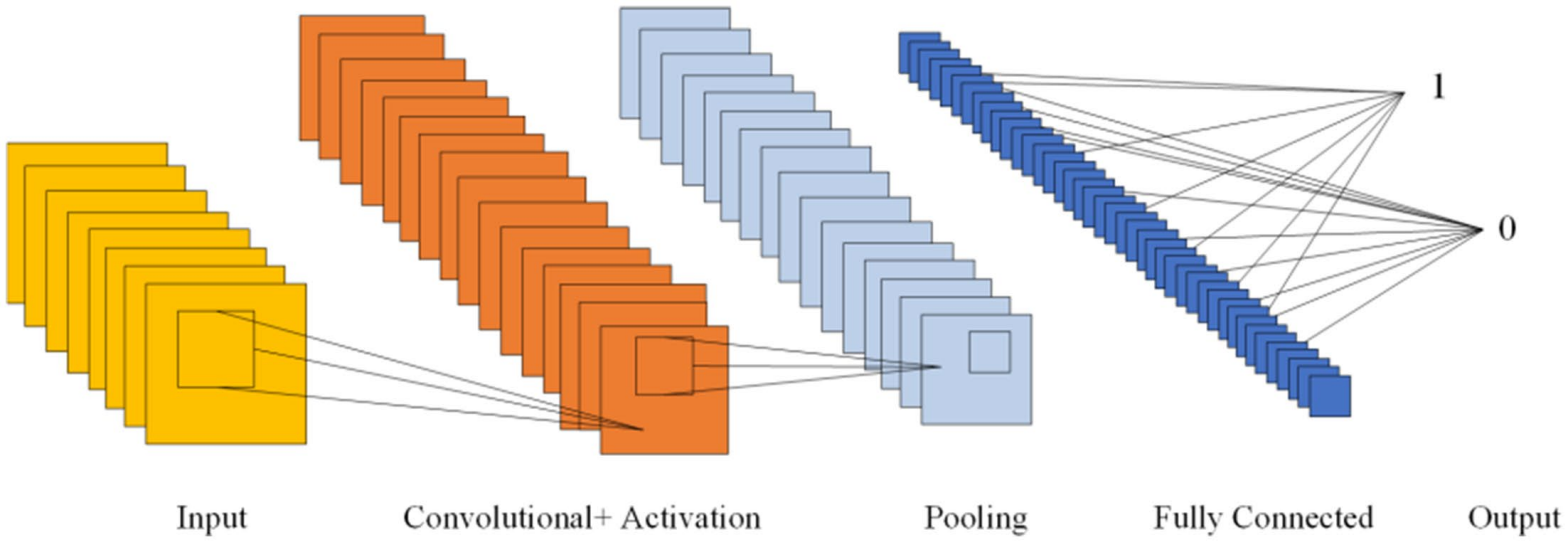
Diagram of CNN.

CNNs, first introduced in 1980, can learn both feature extraction and classification layers within the network. This dual learning enhances the network’s generalization ability, making CNNs particularly effective for processing complex signals (Hassan et al., 2024). Given the non-linear and intricate nature of EEG signals, CNNs are well-suited for their analysis (Hassan et al., 2024). CNNs are structured with several key components: an input layer, convolutional layers, pooling layers, fully connected layers, and an output layer. The convolutional layers, equipped with convolutional kernels, are crucial for extracting features from the input data. Pooling layers further compress the feature space, which reduces computational complexity. The fully connected layers consolidate these features and serve as classifiers. This architecture allows CNNs to be both efficient in computation and effective in training due to the localized and globally shared connections between neurons in the convolutional layers. Figure 6 shows the Diagram of CNN.

Several studies have explored CNNs for epilepsy detection. Acharya et al. (2018) introduced a 1-Dimensional Convolutional Neural Network (1D-CNN) that directly classifies EEG signals without prior feature extraction. Their approach achieved an average accuracy of 88.7%, with a sensitivity of 95% and specificity of 90%. In contrast, Wang et al. (2021) used a stacked 1D-CNN on two public datasets, also skipping pre-processing and feature extraction. Their results demonstrated high accuracy and specificity (over 99%), but the absence of a test set leaves their findings open to debate. Avcu et al. (2019) utilized a 2-Dimensional Convolutional Neural Network (2D-CNN) with only two channels of data, outperforming models that used 18 channels. Das et al. (2024) compared 1D-CNN and 2D-CNN by reshaping data into both formats, finding that the 2D-CNN offered superior classification performance. Pan et al. (2022) proposed a lightweight 2D-CNN that processed both raw data and data transformed by various techniques, such as Discrete Fourier Transform (DFT), Short-Time Fourier Transform (STFT), DWT. While this hybrid method improved detection performance with limited data, the lack of a test set data raised concerns about model reliability. Xu et al. (2024) introduced a 3-Dimensional Convolutional Neural Network (3D-CNN) aimed at reducing the latency of seizure detection through probabilistic prediction, achieving detection delays at least 50% shorter than previously reported.

Table 5 lists various epilepsy detection models based on CNNs, detailing their accuracy (Acc), sensitivity (Sen), and specificity (Spe). The table also indicates the data partitioning methods used, with #1 indicating a segment-based data partitioning approach, #2 indicating an event-based data partitioning approach, *1 indicating an all-patient-based data partitioning approach. If the symbols (#1,#2,*1) are not indicated, it means that the method of partitioning the dataset is not applicable or not specified in the article.

**Table 5:** Epilepsy detection model based on convolutional neural network.

Author	Preprocessing	Feature extraction	Net	Data set	Acc	Sen	Spe
Acharya et al. (2018)	–	–	1D-CNN	Bonn	88.7%	95%	90%
Ullah et al. (2018)	–	–	P-1D-CNN	Bonn	99.1%	–	–
Craley et al. (2019)	Lowpass, Highpass, Notch	Spectral features Line-length	PGM-1DCNN	CHB-MIT	–	61%	–
				Non-public	–	45%	–
^#1^Wang et al. (2021)	–	–	Stacked 1D-CNN	SWEC-ETHZ	99.73%	90.06%	99.81%
				CHB-MIT	99.54%	88.14%	99.62%
Avcu et al. (2019)	–	–	2D-CNN	Non-public	–	93.3%	–
Chen et al. (2023)	Bandpass	DWT, Entropy, Variance	2D-CNN	Bonn	99.9%	100%	99.8%
^*^^1^Das et al. (2024)	EMD	FI, Difference,SODP	2D-CNN	CHB-MIT	99.78%	–	–
Pan et al. (2022)	–	DFT, STFT, DWT	Lightweight 2D-CNN	Bonn	97.89%	–	–
^#2^Xu et al. (2024)	–	STFT	3D-CNN	CHB-MIT	–	94.95%	–
				SWEC-ETHZ	–	94.38%	–

CNNs prediction based on metrics of common classification methods: Shafiezadeh et al. (2024) introduced a calibration pipeline using a 2D-CNN model. By incorporating a small amount of data from test set patients into the training set, their approach fine-tunes the model to predict epileptic events from new patients. This method improved accuracy, sensitivity, and specificity by over 15% on the CHB-MIT dataset and by more than 19% on a non-public dataset. But the computational complexity is too high. Zhang et al. (2021) Proposed Lightweight-2D-CNN reduces computational complexity.

Table 6 outlines various CNNs prediction models and their performance metrics, including accuracy (Acc), sensitivity (Sen), specificity (Spe), and pre-epileptic time (PT) in minutes. It also denotes data partitioning methods: #2 indicating an event-based data partitioning approach, *1 indicating an all-patient-based data partitioning approach, and *2 indicating a cross-patient data partitioning approach. * indicates that the article only describes how the data are divided for non-independent patients and does not mention a specific way of partitioning the data. If the symbols (#2,*1,*2,*) are not indicated, it means that the method of partitioning the dataset is not applicable or not specified in the article.

**Table 6:** CNN prediction model based on common classification method metrics.

Author	Preprocessing	Feature extraction	Net	Data set		Acc	Sen	Spe	PT
^*1*2^Shafiezadeh et al. (2024)	Highpass, Lowpass, Notch	-	2D-CNN	CHB-MIT	*1	82.17%	85.8%	74.02%	15
					*2	69.35%	69.74%	69.90%	
				Non-public	*1	93.78%	93.61%	93.58%	
					*2	70.67%	75.37%	71.28%	
^*^Zhang et al. (2021)	-	Autocorrelation	Lightweight-2D-CNN	CHB-MIT		89.98%	92.9%	87.04%	15
^#2^Jana and Mukherjee (2023)	-	-	NSGA-II+CNN	CHB-MIT		96.51%	96.55%	96.47%	10
^*1*2^Jemal et al. (2024)	Notch, Bandpass	-	CDAN	Siena	*1	96.01%	97.24%	94.57%	60
					*2	60.27%	-	-	
				CHB-MIT	*1	97.36%	98.31%	96.97%	
					*2	70.90%	-	-	

CNNs prediction model based on evaluation criteria for epilepsy prediction: Truong et al. (2018) proposed a 2D-CNN model tested on both scalp and intracranial EEG data. Their model achieved a sensitivity greater than 89% across both datasets, though the Freiburg dataset exhibited a higher FPR. Ra and Li (2023) utilized Simultaneous Extractive Transform (SET) and Singular Value Decomposition (SET-SVD) to enhance time-frequency resolution, achieving a sensitivity over 99.7% with a 1D-CNN model. Ozcan and Erturk (2019) explored spatiotemporal correlations in EEG signals using a 3D-CNN, which showed an 85.7% sensitivity and an FPR of 0.096/h on the CHB-MIT dataset. To improve interpretability: Prathaban and Balasubramanian (2021) developed a dynamic learning framework with a 3D-CNN model optimized by the Fletcher Reeves algorithm. Their phase-transform-based method demonstrated precise real-time seizure prediction using scalp EEG data.

Table 7 presents CNN prediction models based on evaluation criteria such as sensitivity (Sen), FPR, SOP, and SPH, all measured in minutes. It also denotes data partitioning methods: # indicates that the article only describes how the data are divided for independent patients and does not mention a specific way of partitioning the data. If the # is not indicated, it means that the method of partitioning the dataset is not applicable or not specified in the article.

**Table 7:** CNN prediction model based on evaluation criteria for epilepsy prediction.

Author	Preprocessing	Feature extraction	Net	Data set	Sen	FPR/h	SPH	SOP
^#^Ra and Li (2023)	–	SET, SVD	1D-CNN	CHB-MIT	99.71%	–	10	–
				Bonn	100%	–		
^#^Truong et al. (2017)	Bandpass	STFT	2D-CNN	CHB-MIT	81.2%	0.16	5	30
				Freiburg	81.4%	0.06		
				Kaggle	82.3%	0.22		
^#^Truong et al. (2018)	Bandpass	STFT	2D-CNN	Freiburg	89.8%	0.17	5	30
				CHB-MIT	89.1%	0.09		
^#^Ozcan and Erturk (2019)	–	Spectral band power, Statistical moments, Hjorth parameters	3D-CNN	CHB-MIT	85.7%	0.096	1	30
Prathaban and Balasubramanian (2021)	SER	–	3D-OCNN	CHB-MIT	Average: 99.25%	0.045	68.89	–
				Siena		0.05	68.19	–
				Non-public		0.125	62.48	–

### 5.3 Recurrent neural networks

**Figure 7:**
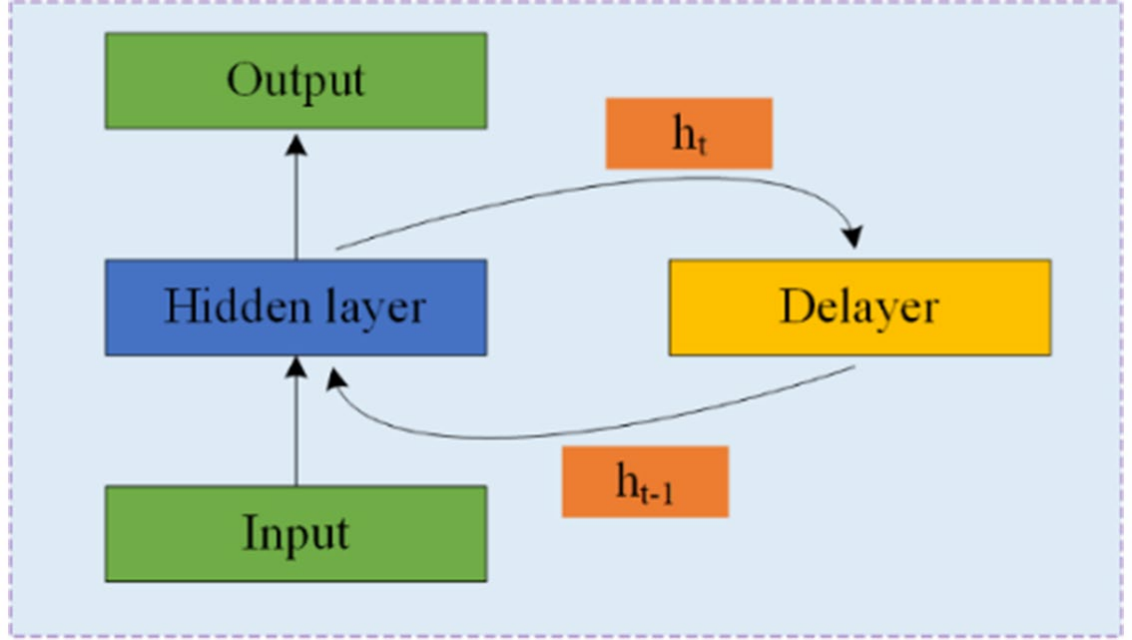
Structure and schematic diagram of RNNs.

RNNs are designed to process time series data and consist of an input, hidden, and output layer. Unlike CNNs, RNNs allow neurons to receive information not only from other neurons but also from themselves, creating a network with loops. This loop structure enables RNNs to maintain a memory of previous inputs through hidden units, making them particularly suitable for processing EEG data. Figure 7 illustrates the structure of an RNN, where *ht* represents the hidden state at time *t*, and the delay mechanism records the most recent hidden state. However, RNNs often struggle with the issue of vanishing gradients, which is mitigated by LSTM and GRU.

**Figure 8:**
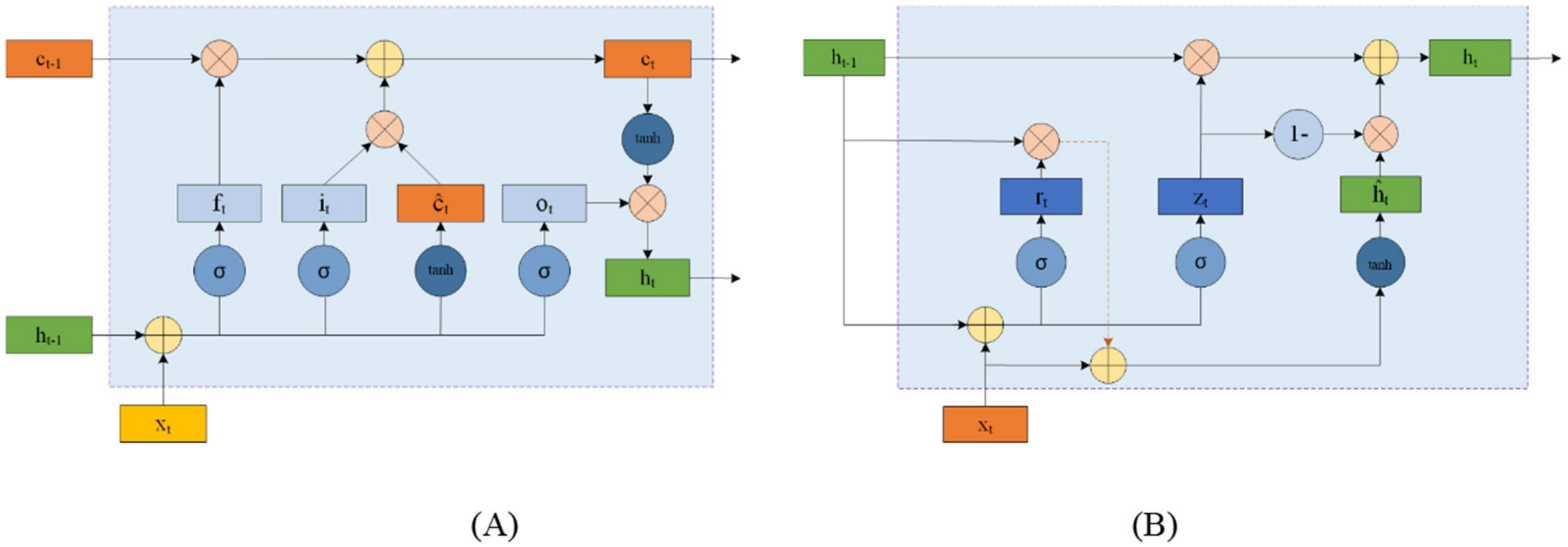
Circular unit structure of LSTMs (A) and GRUs (B).

LSTM networks address this issue by using input and forget gates to manage memory. As shown in Figure 8A, LSTM cells use the external state *h_t − 1_* at time *_t − 1_* and the input *x_t_* at time *t_t_* to calculate the forget gate *f_t_*, input gate *i_t_*, output gate *o_t_*, and candidate state *ĉ_t_*. These elements are then combined with the memory unit *c_t − 1_* to update the memory unit *c_t_* at time *t_t_.* Finally, the external state *h_t_* at time *t_t_* is derived from the memory unit *c_t_* and the output gate *o_t_*. While the complementary relationship between the forget and input gates enhances the LSTM’s memory management, it also introduces some redundancy.

GRU offers a simpler alternative to LSTM by consolidating the input and forget functions into a single gate. Figure 8B shows the structure of a GRU cell, where the reset gate *r_t_* controls the influence of the previous state *h_t − 1_* on the candidate state *ĥ_t_*. The update gate *z_t_* regulates the balance between retaining information from the historical state and incorporating new information from the candidate state. *x_t_* represents the input at time *t_t_*, and *h_t_* is the resulting state.

For epilepsy detection using RNNs, Hu et al. (2020) proposed a Bi-directional Long Short-Term Memory Network (Bi-LSTM) that classifies EEG data using 11 time-domain features extracted through Local Mean Decomposition (LMD). Their method achieved an average sensitivity of 93.61% and specificity of 91.85%. Zhang et al. (2022) also employed a Bi-directional Gated Recurrent Unit (Bi-GRU) for epilepsy detection. They utilized DWT and wavelet energy features, achieving an average sensitivity of 93% and specificity of 98.49%. Tuncer and Bolat (2022) used only instantaneous frequency and spectral entropy features with a Bi-LSTM network, obtaining an average classification accuracy of 97.78%, surpassing Hu’s results but using the Bonn dataset.

In terms of epilepsy prediction, Singh and Malhotra (2022) used a Two-Layer Long Short-Term Memory Network (2 L-LSTM), incorporating Fast Fourier Transform (FFT), spectral power, and mean spectral amplitude for feature extraction. Tsiouris et al. (2018) employed a range of features, including statistical moments, time-domain features, and various transforms (FFT, SD, DWT), within a 2 L-LSTM framework. Their approach demonstrated sensitivity and specificity greater than 99%, outperforming Kuldeep’s results.

### 5.4 Generating adversarial networks

**Figure 9:**
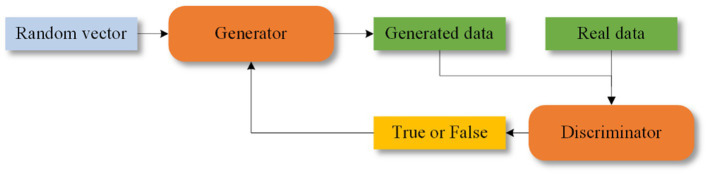
Structure of GANs.

GANs were proposed by Goodfellow et al. (2020). GANs consist of two components: a generator and a discriminator. The generator creates synthetic data, which is then evaluated by the discriminator to determine its authenticity. Feedback from the discriminator helps both components improve their performance. The structure of GANs is shown in Figure 9. Some of the epileptic EEG datasets have less amount of data, e.g., New Delhi dataset, Bonn dataset, this situation leads to poor learning ability of the network and risk of overfitting. GANs are capable of generating simulated data that is very similar to the real data compared to the other data enhancement methods, which improves the performance of the model. GANs have recently been explored for epilepsy classification tasks.

Truong et al. (2019) were among the first to apply GANs to seizure prediction. They used a Deep Convolutional Generative Adversarial Network (DCGAN) to learn features from Time-Frequency domain signals processed with STFT. Despite their innovative approach, DCGAN is known for issues with gradient vanishing and instability (Fathallah et al., 2023).

### 5.5 Transfer learning

Transfer learning can be traced back to the late 20th century and the early 21st century (Burrello et al., 2019; Pan and Yang, 2009). Transfer learning is the use of knowledge gained from one task to improve performance on a related task. First, a basic network is trained on the source dataset and task, and then the learned features (network weights) are applied to a new network trained on a different but related dataset and task. This approach helps to address the problem of EEG data scarcity, and compared to other network models, transfer learning can reduce the resources and time required to train deep learning models (Yang et al., 2024; Weiss et al., 2016; Butt et al., 2024)

In the context of epilepsy detection and prediction, Liang et al. (2020) utilized a combination of the Visual Geometry Group network (VGG) and LSTM for analyzing EEG signals. Their method demonstrated strong results through cross-validation, though sensitivity for some patients was as low as 60%. Gao et al. (2020) employed three deep convolutional neural networks—Inception-ResNet-v2 (Inception Residual Network Version 2), Inception-v3 Network (Inception-v3), and Residual Network 152 (ResNet152)—to classify EEG data into various categories: interictal, reictal I, reictal II, and postictal. They set preictal I and II durations to 30 min and 10 min, respectively, to assess the importance of event duration. Takahashi et al. (2020) proposed an Autoencoder-assisted VGG Network (AE-VGG-16) for seizure detection, which significantly reduced FPR. Toraman (2020) compared three pre-trained CNN models: VGG16, Residual Network (ResNet), and Densely Connected Network (DenseNet)—using spectrogram images to differentiate between pre-seizure and inter-seizure states. The study found that the ResNet model provided the best performance.

### 5.6 Fusion model

**Figure 10:**
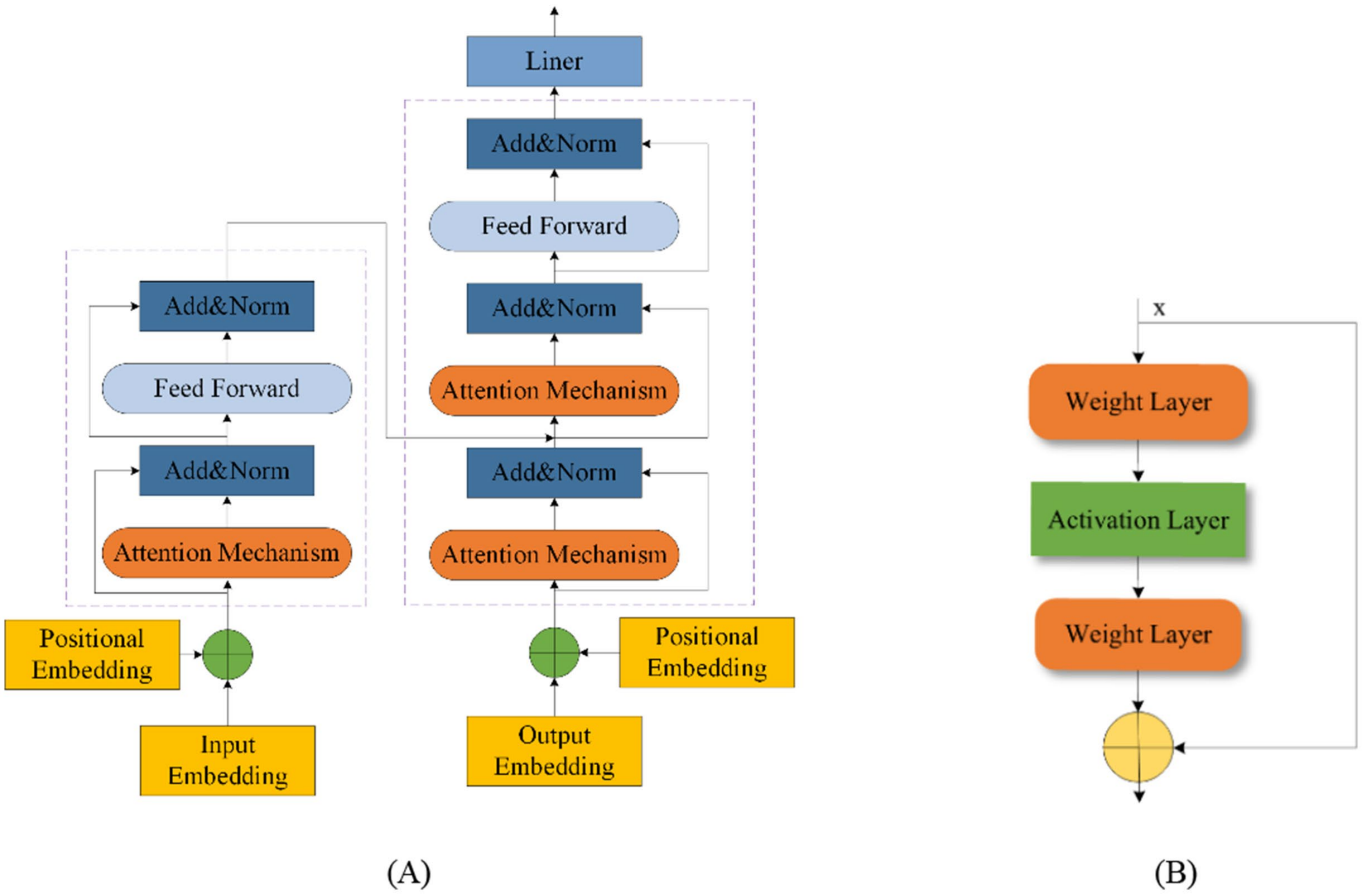
Transformer structure (A) and residual block structure (B).

Fusion modeling combines different network types to leverage their strengths and mitigate their weaknesses. This approach enhances model performance by integrating various methodologies. Transformers and Residual blocks are commonly used in fusion models. The Transformer was first proposed in 2014 by Bahdanau (2014). A Transformer Network includes an input layer, encoder, decoder, and output layer, with a multi-head attention mechanism added between the encoder and decoder (Wang et al., 2024; Zhang et al., 2024a; Cho et al., 2014). This mechanism effectively manages the performance of the model as EEG sequence length increases and improves processing efficiency for long sequences, thus reducing time complexity (De Santana Correia and Colombini, 2022; Rukhsar and Tiwari, 2023). Figure 10A shows the Transformer structure. The ResNet was introduced in 2015 by He et al. (2016). The residual block helps to retain the data from the previous layers and solve problems such as overfitting of the EEG signal on other networks and gradient vanishing. Figure 10B shows the residual block structure.

In the field of epilepsy detection, Sunaryono et al. (2022) introduced a hybrid model combining a 1D-CNN with majority voting and Deep Neural Networks (DNNs). This method achieved 100% accuracy in detecting epilepsy from EEG signals, though its effectiveness may vary with datasets other than the University of Bonn dataset. Awais et al. (2024) proposed a Graph Convolutional Neural Network-Long Short-Term Memory Network (GCN-LSTM), which takes into account the spatial aspects of EEG signals. Huang et al. (2024) developed a Temporal Convolutional Neural Network with a Self-Attention (TCN-SA) layer to extract crucial features using self-attention. Zhu et al. (2024) introduced a Squeeze-and-Excitation Temporal Convolutional Network with Bidirectional Gated Recurrent Units (SE-TCN-Bi-GRU) that automatically selects important EEG channels, though it involves multiple parameters. Zhou et al. (2024) proposed a Lightweight Multi-Attention EEG Network (LMA-EEGNet) that reduces parameter count and network complexity while maintaining effective feature extraction.

Table 8 summarizes various detection algorithms based on hybrid models, detailing their accuracy (Acc), sensitivity (Sen), and specificity (Spe). Data partitioning methods are indicated, with #1 indicating a segment-based data partitioning approach, #2 indicating an event-based data partitioning approach, *2 indicating a cross-patient data partitioning approach. # indicates that the article only describes how the data are divided for independent patients and does not mention a specific way of partitioning the data; * indicates that the article only describes how the data are divided for non-independent patients and does not mention a specific way of partitioning the data. If the symbols (#1,#2,*2,#,*) are not indicated, it means that the method of partitioning the dataset is not applicable or not specified in the article.

**Table 8:** Detection algorithms based on hybrid models.

Author	Preprocessing	Feature extraction	Net	Data set		Acc	Sen	Spe
Sunaryono et al. (2022)	-	DFT, DWT	CNN-DNN	Bonn		100%	-	-
^#^Hussain et al. (2021)	-	FFT, DWT	CNN-LSTM	Freiburg		99.19%	99.3%	98.93%
^*2^Yang et al. (2021)	-	-	CNN-LSTM	Non-public (Video data)		-	88%	92%
Mallick and Baths (2024)	Butterworth		CNN-Bi-LSTM	Bonn		>99%		
^#1#2^Li et al. (2024)	Butterworth	-	CNN-BiLSTM-SCL	CHB-MIT	#1	97.36%	98.97%	97.36%
					#2	-	99.71%	-
^#*^Awais et al. (2024)	-	Statistical moments, Entropy	GCN-LSTM	CHB-MIT	#	96.54%	91.61%	99.15%
					*	99.73%	98.56%	98.74%
^#^Jibon et al. (2024)	Bandpass	Line length, Autocorrelation, Periodogram	SGCN-DeepRNN	TUH-EEG		98.08%	95.13%	94.99%
				CHB-MIT		99.01%	98.06%	95.03%
Wang et al. (2024)	-	FFT, STFT	ID-CNN+2D-CNN+LSTM	Bonn		99.69%	-	-
				New Delhi		97.5%	-	-
Huang et al. (2024)	Highpass,Lowpass, Bandpass	-	TCN-SA	Non-public		95.50%	91.22%	98.72%
				Bonn		97.37%	94.88%	99.91%
^#*2^Zhu et al. (2024)	DWT	-	SE-TCN-Bi-GRU	CHB-MIT	#	98.77%	95.88%	99.44%
					*2	93.78%	93.31%	92.65%
	FIR filter, DWT			Non-public	#	93.61%	94.1%	91.62%
					*2	91.37%	84.85%	93.16%
Wen and Zhang (2018)	-	-	AE-1D-CNN	Non-public		95.26%	-	-
^#^Yang et al. (2024)	Bandstop, PCA	-	LTY-CNN	Non-public		99.9%	99.9%	98.8%
				CHB-MIT		99.0 %	99.1%	93.2%
Zhou et al. (2024)	Bandpass	PSD	LMA-EEGNet	Helsinki		93.29%	94.43%	92.14%
Zhao et al. (2024)	-	-	Res-BiLSTM	Bonn		98.88%	-	-
				TUH		95.03%	-	-
Li et al. (2023)	Butterworth	STFT	ResNet-BiGRU	Non-public		92%	92.43%	99.99%

Fusion Model Predictions Based on Common Classification Indicators: Aslam et al. (2022) employed a Convolutional Neural Network combined with Long Short-Term Memory (CNN-LSTM) to classify EEG signals and predict seizures. This approach yielded effective results in classifying temporal sequences. To enhance the handling of long-term temporal dependencies, Indurani and Vandana (2023) utilized a Time-Attention Convolutional Neural Network with a Recurrent Neural Network (TA-CNN-LSTM). Meanwhile, Ma et al. (2023) introduced a Convolutional Bi-directional Long Short-Term Memory Network model based on Multi-Channel Feature Fusion (MCFF-CNN-Bi-LSTM). This model, which integrates an attention mechanism and channel fusion, effectively manages long-term temporal signals while reducing computational complexity.

Table 9 summarizes various hybrid models evaluated using predictive and categorical assessment methods. Accuracy (Acc), sensitivity (Sen), and specificity (Spe) are the primary metrics. Data partitioning methods are indicated, with #1 indicating a segment-based data partitioning approach, #2 indicating an event-based data partitioning approach, and *2 indicating a cross-patient data partitioning approach. # indicates that the article only describes how the data are divided for independent patients and does not mention a specific way of partitioning the data; * indicates that the article only describes how the data are divided for non-independent patients and does not mention a specific way of partitioning the data. If the symbols (#1,#2,*2,#,*) are not indicated, it means that the method of partitioning the dataset is not applicable or not specified in the article.

**Table 9:** Hybrid models based on predictive and common assessment methods.

Author	Preprocessing	Feature extraction	Net	Data set	Acc	Sen	Spe	PT
^#^Usman et al. (2020)	Butterworth, Bandpass	STFT	CNN-SVM	CHB-MIT	–	92.7%	90.8%	21
^*^Hu et al. (2023)	–	Amplitude spectrum	CNN-SVM	CHB-MIT	86.25%	–	–	60
Aslam et al. (2022)	Highpass: REP Pipeline, STFT	Statistical moments	CNN-LSTM	CHB-MIT	94%	93.8%	91.2%	19.5
^#1^Wei et al. (2019)	ICA	–	LRCN	Non-public	93.40%	91.88%	86.13%	30
^*^^2^Choi et al. (2022)	Highpass, Lowpass, Notch	–	ACGRU	Non-public	82.86%	80%	85.5%	10
^#2^Daoud and Bayoumi (2019)	–	–	DCAE -Bi-LSTM-CS	CHB-MIT	99.66%	99.72%	99.60%	60
Indurani and Vandana (2023)	–	–	CGAN- TACNN-LSTM	CHB-MIT	94.6%	94.5%	–	–
				Bonn	94.8%	94.9%	–	
Ma et al. (2023)	–	–	MCFF-CNN-Bi-LSTM	CHB-MIT	94.83%	94.94%		<0.01
				Bonn	77.62%	77.62%		

Fusion Model Predictions Based on Epilepsy-Specific Evaluation Criteria: Wei et al. (2019) used a CNN-LSTM model to predict epileptic seizures, focusing on its effectiveness in handling EEG signals. Yan et al. (2022) proposed a three-transformer tower model to address the spatial and temporal characteristics of EEG signals. However, this model may suffer from parameter redundancy and insufficient representation of spatial structures. Liu et al. (2024) introduced a Pseudo 3D-CNN combined with Directed Convolutional Long Short-Term Memory (P3D BiConvLstm3D), which employs Max-Relevance and Min-Redundancy (mRMR) and a 3D channel attention mechanism to enhance feature selection and reduce redundancy.

Table 10 provides a summary of hybrid models based on predictive and epilepsy-specific evaluation criteria. Sensitivity (Sen), FPR, SOP, SPH are key metrics. Data partitioning methods are indicated, with #1 indicating a segment-based data partitioning approach, #2 indicating an event-based data partitioning approach. # indicates that the article only describes how the data are divided for independent patients and does not mention a specific way of partitioning the data.

**Table 10:** Hybrid model based on predictive and epilepsy prediction evaluation criteria.

Author	Preprocessing	Feature extraction	Net	Data set	Sen	FPR/h	SPH	SOP
^#2^Wei et al. (2019)	ICA	–	LRCN	Non-public	91.88%	0.04	5	30
^#2^Sun et al. (2021)	Bandpass	STFT	CADCNN	CHB-MIT	97.1%	0.029	3	30
^#^Ji et al. (2023)	Bandpass	STFT	GAM-BiGRU	CHB-MIT	88.09%	0.053	5	30
^#^Rasheed et al. (2021)	Butterworth	STFT	DCGAN+CNN+transfer learning	CHB-MIT	88.21%	0.03	10	30
^#1^Yan et al. (2022)	Bandpass	STFT	3 Transformers	CHB-MIT	96.01%	0.047	3	30
^#2^Shi and Liu (2023)	Bandpass	STFT	B2-ViT	CHB-MIT	93.3%	0.057	5	30
				Kaggle	85.2%	0.013		
^#^Liu et al. (2024)	Bandpass, ICA	FuzzyEn	P3D-BiConvLstm3D	CHB-MIT	98.13%	–	5 ~ 15	15

Ngiam et al. (2011) pioneered the use of deep learning techniques to integrate and learn from multiple types of data. Multimodal learning in deep learning extends beyond EEG signals, incorporating various data types to enhance model performance in real-time epilepsy detection. By integrating diverse data sources, these models achieve more accurate and comprehensive results.

Ahmedt-Aristizabal et al. (2018) utilized a CNN-LSTM model to combine features from facial, body, and hand movements, effectively distinguishing between Mesial Temporal Lobe Epilepsy (MTLE) and Extratemporal Lobe Epilepsy (ETLE). This approach highlights the effectiveness of incorporating multiple types of input data in improving diagnostic accuracy. Sirpal et al. (2019) integrated Functional Near-Infrared Spectroscopy (fNIRS) with EEG in a multimodal EEG-fNIRS-LSTM-RNN model, achieving up to an 8% improvement in classification accuracy. This model demonstrates the benefits of combining different data modalities for enhanced seizure detection. Hosseini et al. (2020) proposed a model combining Long Short-Term Memory-Support Vector Machine (LSTM-SVM) with edge computing, using both resting state-functional Magnetic Resonance Imaging (rs-fMRI) and EEG data. This approach allows for autonomous localization of epileptic foci and improved prediction capabilities. Martini et al. (2021) employed Stereo Electroencephalogram (SEEG) and video electroencephalogram data in a self-supervised LSTM-based network. This model’s use of self-supervised dynamic thresholding enhanced robustness and efficiency, significantly improving sensitivity in real-time epilepsy prediction. Aluvalu et al. (2024) introduced a Multimodal Convolutional Neural Network (MMFCNN) utilizing a cross-attention mechanism. By filtering data through a cognitive framework and analyzing patient movements, gestures, and facial expressions, the MMFCNN achieved an impressive accuracy rate of 99.2%.

## 6 Discussion

Among the publicly available datasets for epilepsy detection and prediction, the CHB-MIT dataset is the most widely used. This preference is due to several advantages of the CHB-MIT dataset: Large Volume of Data: CHB-MIT provides a substantial amount of data, which supports effective model training. Flexible Data Structure: The dataset is organized by patient, with each patient’s data stored in separate folders. It includes continuous recordings of both seizure and non-seizure phases, making it suitable for various analytical needs. Researchers can utilize the data for both detection and prediction tasks, with the flexibility to employ both patient-independent and non-patient-independent data partitions. Additionally, it allows for customization of seizure periods, inter-ictal, and pre-ictal phases, enabling more precise model analysis.

Despite its benefits, the CHB-MIT dataset, like other publicly available datasets, has notable limitations. The scope of epilepsy datasets is limited, and the data are collected under controlled conditions that may not capture the full complexity of real-life epilepsy occurrences. For instance, epilepsy can manifest during diverse activities such as walking, eating, or exercising, where EEG signals can be highly variable. Current public datasets may not fully address these real-world scenarios, leaving gaps in the comprehensiveness of available data.

Most research in the field employs patient-independent data partitioning, with only a few studies using non-patient-independent methods. While non-patient-independent partitioning is less common, it is an essential approach for certain analyses. Additionally, some studies do not clearly specify their data segmentation methods, which can lead to confusion among readers. This paper argues that explicitly stating the data segmentation approach is crucial for enhancing the clarity and understanding of the literature.

EEG preprocessing and data enhancement methods vary widely in the literature. Many researchers employ basic filtering techniques for EEG signals. The artifact removal methods such as WT, EMD, and BSS can improve the data quality, but they require the use of reasonable thresholds. Some researchers, on the other hand, have achieved satisfactory classification results by using raw data directly. This practice may be dictated by the nature of overt epileptic EEG signals, which are usually collected under controlled conditions where subjects try to remain as motionless as possible, making the partial noise very faint. However, real-world conditions differ, as epileptic patients may not stay stationary. Data augmentation using GANs involves complex training processes with high computational demands. A more accessible approach is window overlapping, which segments data to enhance its volume effectively. Combining multiple publicly available EEG datasets is also common, as it not only increases the volume of data but also demonstrates the model’s generalization capability and reliability.

Given the strengths and limitations of various feature extraction methods, feature fusion techniques have become popular. Feature fusion combines EEG features of different dimensions to provide a more comprehensive view of the data. However, an excessive number of features can lead to information redundancy. To address this issue, several feature selection methods have been proposed, including the Fisher score, Group Search Algorithm, Crow Search Algorithm, and Optimal Feature Selection Algorithm (Kapoor et al., 2023; Subasi et al., 2019). Traditional manual feature extraction methods often suffer from low generalization and suboptimal performance. To overcome these limitations, researchers have increasingly integrated manual feature extraction with deep learning techniques (Cao et al., 2019; Yuan et al., 2018). This combined approach leverages the strengths of both methods, enhancing model performance and accuracy in epileptic seizure detection and prediction.

Currently, evaluation criteria for epilepsy detection tasks are relatively standardized, focusing primarily on classification metrics. For epilepsy prediction tasks, evaluation methods are categorized into two main types: those based on classification metrics and those specific to epilepsy prediction. Despite their widespread use, these criteria have notable shortcomings: particularly in the definition and application of the FPR. Most studies define FPR as the number of false positives recorded within an hour of EEG monitoring. However, there is variation in this definition across the literature. For instance, one approach uses confusion matrices to determine FPR, while another defines it as the number of false positives during the event interval (Wei et al., 2018; Khan et al., 2017). This latter method can be problematic because, with varying intervals between episodes, longer intervals result in a lower FPR, while shorter intervals lead to a higher FPR. This inconsistency makes it challenging to compare results across different studies and datasets.

**Figure 11:**
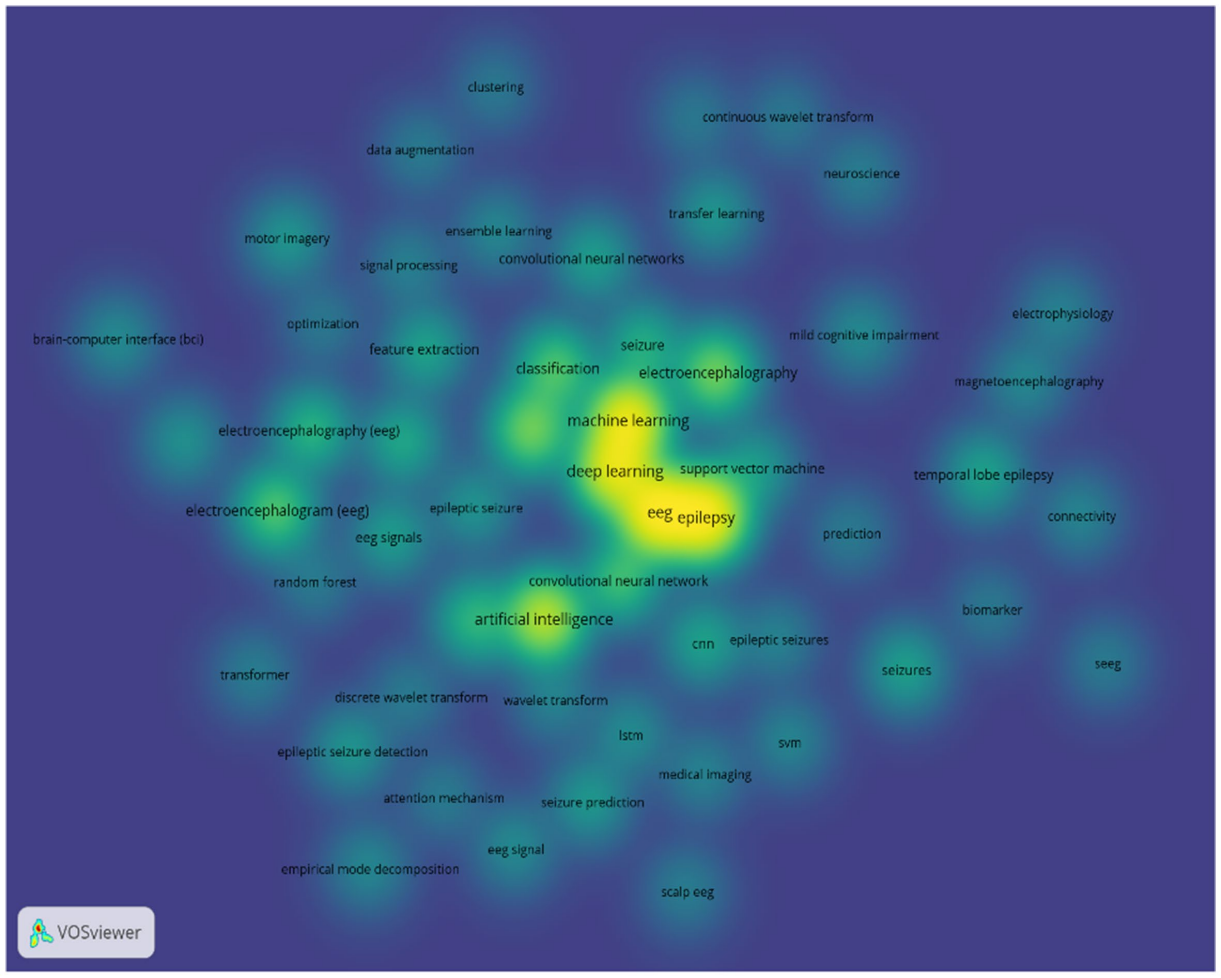
Analysis of machine learning and deep learning methods for EEG-based epilepsy detection and prediction.

Deep learning algorithms for epilepsy detection and prediction have seen a wide range of approaches. For our study, we reviewed more than 2,000 research papers on machine learning and deep learning techniques for detecting and predicting epileptic events using EEG signals. These papers are from ScienceDirect and Springer databases and span from 2020 to 2024. We conducted a keyword visualization analysis of the trends in these papers using VOSviewer software. The results of the analysis show that deep learning, as a subset of machine learning: lays an important role in processing EEG signals for epilepsy detection and prediction, as shown in Figure 11. CNNs are the most commonly used models in this field. In contrast, RNNs, GANs, and transfer learning models are less frequently employed. GANs, initially used primarily for data augmentation, were first applied to epilepsy prediction in 2019, highlighting their potential for further development in this area (Truong et al., 2019). Multimodal deep learning methods offer the advantage of learning features from various perspectives, enabling real-time detection and prediction. However, these methods require substantial data and advanced hardware, which can be limiting. Spiking Neural Networks (SNNs), which model biological neuron dynamics and offer high capacity with low energy consumption, are less commonly used due to their training difficulties (Akopyan et al., 2015; Sengupta et al., 2019). Zhang et al. (2024b) have addressed this issue by introducing a biologically inspired impulse recurrent neural network that performs well in cross-patient epilepsy detection, potentially expanding the practical applications of SNNs. In response to the high memory consumption and redundancy issues associated with traditional CNNs, Liu et al. (2024) proposed a Cosine Convolutional Neural Network (CosCNN) that reduces memory cost by nearly 75% with minimal loss in accuracy. Another innovation, the Capsule Network introduced by Sabour et al. (2017), overcomes the limitation of CNNs in capturing feature relationships and has been successfully applied to epilepsy detection and prediction (Li et al., 2022; Toraman, 2021).

Fusion models, which combine various algorithms, generally yield better results. Attention mechanisms are frequently employed in these models due to their ability to handle long time series data efficiently, making them well-suited for epileptic EEG signals. Combining CNNs with RNNs is also common, leveraging CNNs for feature extraction and RNNs for time series processing to capture both deep features and temporal aspects of EEG data. With the increasing availability of publicly accessible datasets, researchers are now combining multiple datasets to enhance model generalization and recognition. However, it is crucial to differentiate between validation and test sets: validation data can be used for model training, while test data should remain unseen during training to ensure accurate evaluation.

Overall, algorithms for epilepsy detection tend to outperform those for epilepsy prediction. This discrepancy may be attributed to dataset characteristics, such as those found in the CHB-MIT and Freiburg datasets. The fixed seizure periods in detection tasks create a more distinct contrast between ictal and interictal states, making classification easier compared to the subtler differences between pre-ictal and interictal periods.

Evaluation criteria for epilepsy prediction often involve stricter requirements aligned with clinical judgments. Typical pre-seizure windows are defined as less than 1 h, with specific periods such as 1–15 min for SPH and 30 min for SOP. These criteria reflect the challenges of aligning predictive algorithms with practical clinical needs.

## 7 Conclusion

Epilepsy is a chronic neurological disorder that significantly affects both the physical and mental health of patients, potentially leading to conditions such as anxiety and depression (Watanabe et al., 2024). For healthcare providers, manually analyzing a large volume of epileptic EEG signals is extremely challenging, with detection accuracy heavily reliant on individual expertise. As technology advances, employing deep learning techniques for epilepsy.

Significant progress has been made in improving algorithms for preprocessing, feature extraction, and neural networks. Nevertheless, several limitations remain. First, there is a scarcity of diverse, publicly available datasets for different types of epilepsy, with most data collected under specific conditions that may not reflect real-world scenarios. Second, data preprocessing methods are often limited and lack variety. Third, there is inconsistency in the evaluation of FPR. Fourth, no standard evaluation criteria exist for epilepsy prediction models. Fifth, the ability to distinguish between interictal and preictal periods needs further refinement. Sixth, many studies rely on empirical definitions for seizure prediction and evaluation, with inconsistent settings for SPH and SOP. Seventh, high computational and time complexity of current models hampers real-time detection capabilities.

To address these issues, future research should focus on: (1) expanding the dataset to include more diverse epilepsy cases; (2) integrating methods like temporal and feature thresholding to enhance interpretability and segmentation between interictal and preictal periods; (3) utilizing advanced seizure prediction features to optimize parameters such as SPH, SOP, and FPRmax; (4) refining algorithmic models to minimize memory and computational demands, thus facilitating real-time detection; and (5) exploring models from other research areas to enhance the effectiveness of epilepsy detection and prediction systems.
